# *Trichoderma* spp. Improves Flowering, Quality, and Nutritional Status of Ornamental Plants

**DOI:** 10.3390/ijms232415662

**Published:** 2022-12-10

**Authors:** Roman Andrzejak, Beata Janowska

**Affiliations:** 1 Department of Phytopathology, Seed Science and Technology, Faculty of Agronomy, Horticulture and Bioengineering, Poznan University of Life Sciences, Dąbrowskiego 159, 60-594 Poznań, Poland; 2 Department of Ornamental Plants, Dendrology and Pomology, Faculty of Agronomy, Horticulture and Bioengineering, Poznan University of Life Sciences, Dąbrowskiego 159, 60-594 Poznań, Poland

**Keywords:** biostimulant, ornamental plants, flowering, quality, micro- and macroelements

## Abstract

Scientists all over the world conduct research to determine the influence of *Trichoderma* spp. on various groups of plants, mostly crops. However, there is little information on the influence of these fungi on ornamental plants. Therefore, the authors of this study analyzed the influence of *Trichoderma* spp. on the growth, flowering, quality, and nutritional status of ornamental plants. The research showed that *Trichoderma* spp. in this group of plants stimulate the elongation and thickening of shoots and the formation of leaves. These fungi also stimulate or inhibit leaf elongation. They also accelerate the flowering of plants, stimulate the elongation of inflorescence shoots and inflorescences, and the development of flowers. Apart from that, *Trichoderma* spp. positively influence the content of chlorophyll and carotenoids in leaves, and they stimulate the uptake of micro- and macroelements.

## 1. Introduction

A plant biostimulant is any substance or microorganism applied to plants with the aim to enhance nutrition efficiency, abiotic stress tolerance, and/or crop quality traits, regardless of their nutrient content. Many biostimulants improve nutrition and they do so regardless of their nutrient contents. Biofertilizers increase nutrient use efficiency and open new routes of nutrients acquisition by plants. In this sense, microbial biostimulants include mycorrhizal and non-mycorrhizal fungi, bacterial endosymbionts (such as *Rhizobium*), and Plant Growth-Promoting Rhizobacteria (PGPR). Thus, microorganisms applied to plants can have a dual function of biocontrol agent and biostimulant [1]. The biostimulant segment is becoming increasingly important worldwide. One of the reasons for this is that fewer plant protection products are placed on the market in the European Union, and environmental sustainability also plays an important role in their use [2].

Fungi of the *Trichoderma* genus are considered as biostimulants. They are widely distributed in the environment. They are present in all climatic zones and inhabit a variety of ecological niches. The most common habitats for *Trichoderma* spp. include decaying wood, soil, and, above all, the rhizosphere. Fungi of this genus produce numerous metabolites that support their interaction with plants and other microorganisms. *Trichoderma* spp. interact with bacteria, viruses, and pathogenic fungi through hyperparasitism and antibiosis [3]. These fungi have the ability to reduce toxins produced by fungi of the *Fusarium* genus [4,5]. Recently, it has been demonstrated that they may also have complementary properties that strengthen plant defense barriers against insects [6]. However, the use of these fungi is limited, to an extent, by their variable level of biocontrol activity, which is influenced by environmental conditions. *Trichoderma* spp. cause Induced Systemic Resistance (IRS) in monocotyledonous and dicotyledonous plants as a result of biotic and abiotic stress. Owing to these properties, they are classified as Biological Control Agents (BCA) that are used commercially as biopesticides or biostimulants in the production of plant protection products. *Trichdoderma* spp. produce many biologically active compounds, such as enzymes (cellulases, proteases, phosphatases, lipases, xylanases, and amylases) [7], antibiotics, volatile compounds [4,8,9,10], and growth regulators [4,11]. Owing to their properties, *Trichoderma* spp. are included in microbiological formulations used to optimize the composting of materials of various origins [7].

*Trichoderma* spp. are widely described as plant growth stimulators. This trait tends to be isolate-specific rather than species-specific, whereas individual isolates show different degrees of plant specificity. Increased root and/or shoot biomass is the most common manifestation of growth stimulation; however, changes in plant morphology and development have been described too. Growth stimulation can be highly variable due to several limiting factors, such as crop type, conditions, inoculum dose, and type of formulation [7]. According to Nieto-Jacob et al. [12], communication between plants and *Trichoderma* spp. involves the recognition of molecules derived from the fungi, such as auxins and micro-organic Volatile Organic Compounds (VOCs); however, this communication is highly dependent on the environment. Contreras-Cornejo et al. [13] suggest that *Trichoderma* spp. induce growth by an auxin-dependent mechanism. They used in vitro biological tests to demonstrate that *T. virens* Gv29.8 and *T. atroviride* IMI206040 can synthesize indole-3-acetic acid (IAA) and some of its derivatives, which results in vigorous development of the root system. These authors claim that many strains of *Trichoderma* spp. are capable of synthesizing IAA, but only some of them can stimulate plant growth. Some researchers point out that *Trichoderma* spp. stimulate plant growth because they enable plants to absorb more nutrients and support the production of vitamins and growth regulators [14,15,16]. A wide range of *Trichoderma* bioinoculants are currently available on the market. Mixtures of strains are gaining popularity, as they ensure more consistent performance [11]. 

The production of ornamental plants is one of the fastest growing areas in the horticultural sector. Of particular importance is the cultivation of potted ornamental plants, which has an upward trend in the international market worldwide [17]. After the global economic crisis in 2008, the production of ornamental plants slowed down. Today, however, it plays a significant role in the horticultural sector. Ornamental plants are also playing more and more important roles in the urban environment, e.g., reducing air pollution [18]. The global export of ornamental plants is growing, and it reached a value of USD 9.4 billion already in 2014 [19]. The ornamental plant trade has become a leading sector in previously unnoticed countries such as Brazil [20] and Thailand [19]. Moreover, the development of the floriculture sector goes hand in hand with the economic growth of developing countries [21].

Research intended to determine the impact of *Trichoderma* spp. on various groups of plants has been conducted around the world. It has been dominated by research on commercial, edible plants [22,23,24]. However, little information is available on the impact of these fungi on ornamental plants. Therefore, this paper examines the research assessing the impact of *Trichoderma* spp. on the growth, flowering, quality, and nutritional status of ornamental plants.

## 2. Classification and Morphology of *Trichoderma* spp.

The name of the genus *Trichoderma* was introduced into the literature by Persoon in 1794 [25]. The genus belongs to the kingdom of Fungi, phylum Ascomycota, class Sordariomycetes, order Hypocreales, family Hypocraceae [26]. In 1865, the Tulasne brothers proved that *Hypocrea rufa* was a teleomorph of *T. viride* Pers. [27]. Until 1939, it was believed that there was only one species within the genus *Trichoderma*—*T. viride* [28]. Then, in 1969, based on the analysis of morphological features, Rifai [29] distinguished nine species: *T. harzianum* Rifai, *T. viride*, *T. hamatum* (Bonord.) Bainier, *T. koningii* (Oudem.) Duché & R. Heim, *T. polysporum* (Link) Rifai, *T. piluliferum* J. Webster & Rifai, *T. aureoviride* Rifai, *T. longibrachiatum* Rifai, and *T. pseudokoningii* Rifai. In the early 1990s, Bissett [30,31,32] identified five sections and 27 biological species within the genus *Trichoderma*. The introduction of such tools as restriction fragment length polymorphism markers (RFLP), random amplified polymorphic DNA markers (RAPD), and phylogenetic markers of coding sequence variation for the molecular identification of species had a significant impact on the development of taxonomy at that time. From the late 1990s to 2002, the number of *Trichoderma* species increased to 47 [33]. Verification of the taxonomy of the entire genus was initiated by Kindermann et al. [34], who analyzed the sequence of the internal transcribed spacer 1 (ITS1) region that encodes the rRNA. The further development of molecular methods, including the presentation of the first fungal oligonucleotide barcode for the identification of *Hypocrea* and *Trichoderma* species—*TrichO* Key version 1.0 [35], has contributed to doubling of the number of newly described species. Currently, there are 497 species of genus *Trichoderma* listed in Index Fungorum [26]. However, it should be emphasized that the number of the so-called morphological species has not increased dramatically, and amounts to 1/3 of the species described based on molecular analyses [36].

**Figure 1:**
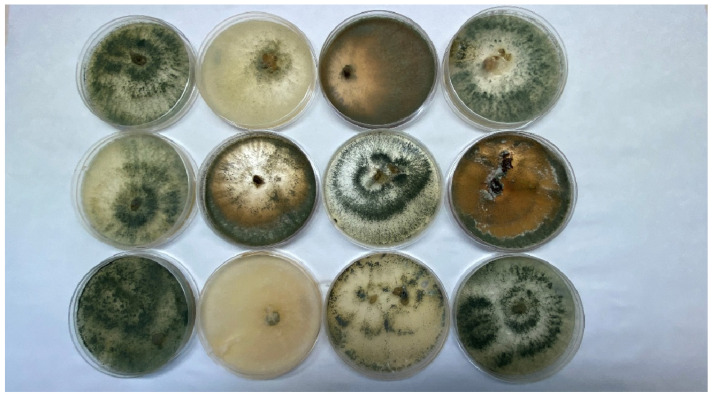
Colonies of fungi of the *Trichoderma* genus.

*Trichoderma* spp. exist in conidial (imperfect) stages, which makes them unable to reproduce sexually, as well as in perfect stages, such as Hypocrea; in which case, sexual reproduction is possible. The germinating spores, or chlamydospores, develop by forming simple or branched conidiophores, the conidial spores of which are spherical or ellipsoidal in shape. Sporulation depends on the availability of nutrients and light, temperature, and competition from other microorganisms [37]. *Trichoderma* spp. form circular conidial zones, formed by fungal colonies made up of bundles of conidiophores, clumped or loose. The surface of the colony resembles cotton wool. The color of the conidia varies from white-green to dark green, depending on the species (Figure 1). Some colonies of *Trichoderma* spp. produce an odor, e.g., *T. atroviride* and *T. harzianum*, which smell like hazelnuts [29].

## 3. The Impact of the Environment on the Population of *Trichoderma* spp.

*Trichoderma* fungi are found in almost all types of soil around the world. The soils in temperate and tropical climates contain between 10^1^ and 10^3^ propagule units of those fungi per 1 g. They inhabit roots of various cultivated and wild plants [13,38].

The carbon(C)-to-nitrogen(N) ratio (C:N) has a great impact on the development of *Trichoderma* spp.—a ratio too low results in the loss of competitive interactions between *Trichoderma* spp. and fungal plant pathogens [39]. This is due to the fact that *Trichoderma* spp. is able to use various sources of C and N to grow. The demand for C and energy is covered by simple and complex sugars, as well as purines, pyrimidines, amino acids, thiamine, alkaloids, and organic acids, especially long-chain fatty acids and even methanol (CH_3_OH) and methylamine (CH_3_NH_2_). The most frequently used source of N is ammonia (NH_3_); however, *Trichoderma* spp. also use amino acids, urea (CO(NH_2_)_2_), nitrites, and nitrates. When the concentration of N in the substrate increases, many isolates respond by forming a mass of conidial spores and chlamydospores [38]. The favorable C:N ratio for *Trichoderma* spp. is found in soils rich in C and phosphorus (P) [40]. The development of *Trichoderma* spp. is also determined by abiotic factors (substrate and air temperature, humidity, substrate pH) and biotic factors (interactions between microorganisms). In unfavorable environmental conditions, e.g., too high temperature, the conidia of *Trichoderma* spp. may die, as their walls are too thin. However, the fungi can survive thanks to the formation of thick-walled chlamydospores. Such a phenomenon is observed in *T. hamatum*, *T. harzianum*, *T. virens*, and *T. viride. Trichoderma* spp. are classified as mesophilic organisms, as the optimum temperature for the growth and development of those fungi is approximately 25 °C [37]. However, some strains of *T. viride* and *T. polysporum* can grow at low temperatures. Additionally, cold-tolerant strains of *T. viride*, *T. harzianum*, and *T. aureoviride* can become parasites of phytopathogens such as *Rhizoctonia solani*, *Fusarium oxysporum* f. sp. *dianthi* at low temperatures (5–10 °C) by producing enzymes characteristic of the mycoparasitism: β-glucosidase; β-1,4,-N-acetylglucosaminidase; or trypsin and chemotrypsin proteases [37].

*Trichoderma* spp. grow very fast when the pH of the substrate is 5–5.5, but they are easily decomposed in the light, as they are sensitive to UV radiation [37]. According to Benitez et al. [4], the development of *Trichoderma* spp. also takes place in alkaline substrate, with a large amount of carbon dioxide (CO_2_). Das et al. [41] indicate that high humidity (80%) is very important for those fungi to develop properly.

Copper (Cu) ions can also affect the growth rate, sporulation, and enzymatic activity of *Trichoderma* spp. [42].

*Trichoderma* spp. show high resistance to many toxic compounds produced by other microorganisms, including antibiotics, as well as to terpenoid phytoalexins and peroxidases secreted by plants, and to fungicides and heavy metals. Based on molecular studies, the resistance that makes those fungi active colonizers and strong competitors may be related to the ability of *Trichoderma* spp. to produce ABC protein transporters [43]. Those proteins have the adenosine 5’-triphosphate-binding cassette (ATP). The energy released by them as a result of ATP hydrolysis is used to transport various types of substrates across the membrane or for processes not related to transport, such as RNA translation and DNA repair [44]. As a result of overexpression of ABC transporter genes, the accumulation of toxins in the cells of *Trichoderma* spp. is limited [43]. 

## 4. Ways to Use *Trichoderma* spp.

*Trichoderma* spp. are currently sold in the form of biopesticdes, biofertilisers, and stimulants for growth and natural resistance. The effectiveness of these fungi can be attributed to their ability to protect plants, stimulate vegetative growth, and restrict the population of pathogens, as well as to act as substrate additives (inoculants) that improve nutrient uptake capacity. Live fungal spores (active substance) are incorporated into a variety of preparations (traditional, as well as innovative) that are used as solutions for spraying on the leaves, on seeds, and on young plants, in post-pruning treatments in the substrate for sowing or transplanting, as well as for watering or soaking of, e.g., spore organs such as tubers, bulbs, and rhizomes. Formulations based on *Trichoderma* spp. are sold across the world and used to protect crops against various plant pathogens and to stimulate the growth and productivity of plants in various growing environments, such as fields, greenhouses, nurseries, and in the production of various horticultural crops, fruit crops, trees, and ornamental plants (Table 1). Most bioproducts with *Trichoderma* spp. are manufactured in Asia, followed by Europe, South-Central America, and North America. Most labels point to the fungicidal properties of these formulations; however, only 38% of the products available on the market have been registered. Ten *Trichoderma* species have been specifically designated for the use on plants representing different groups; yet, many labels indicate that *Trichoderma* spp. are offered as a mixture of different fungi of that genus. The most popular format of these formulations is a dampened powder made from a specific concentration of dried conidial spores of the fungus in the form of fine dust that requires mixing with water. Other common formats are granulated, liquid, and solid formulations [45].

**Figure 2:**
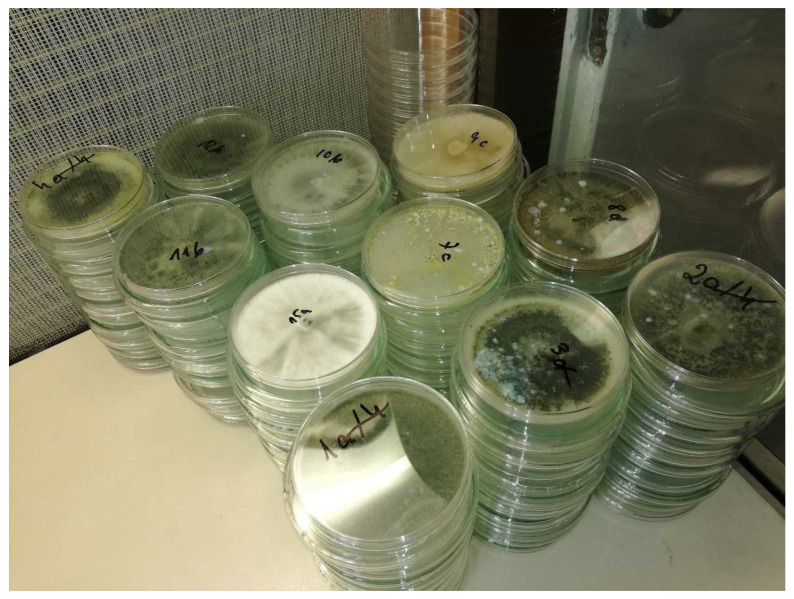
*Trichoderma* spp. isolates on Petri dishes.

**Figure 3:**
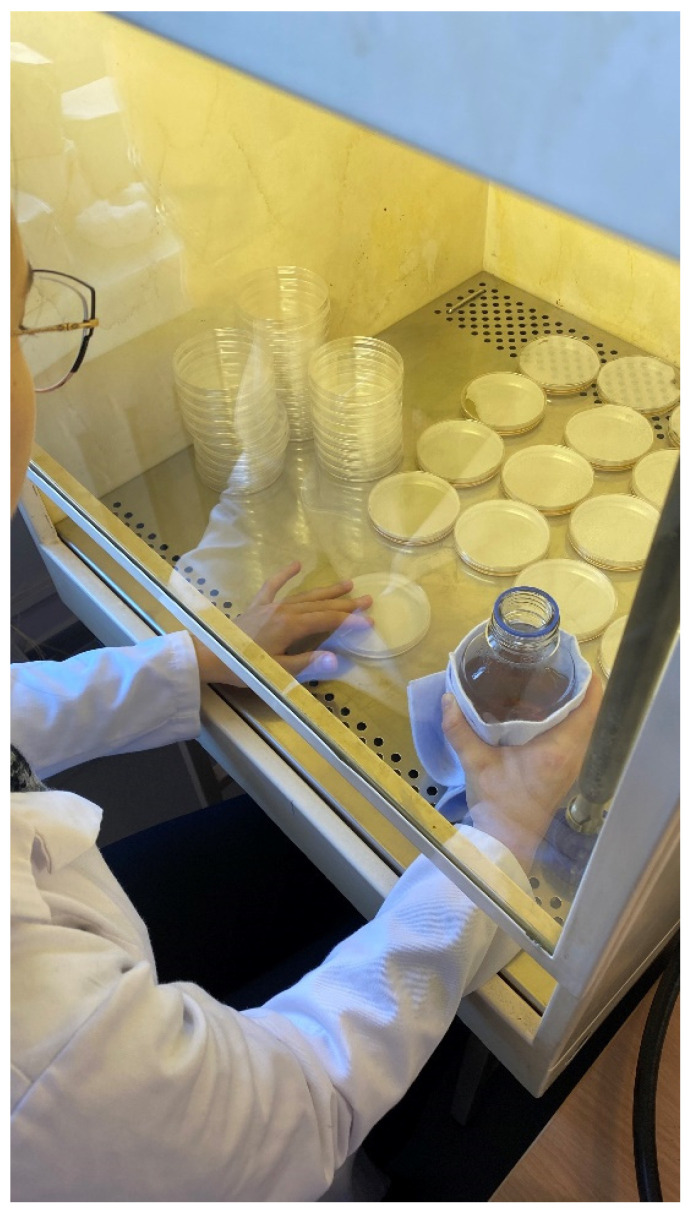
PDA medium in Petri dishes.

Individual *Trichoderma* spp. fungi from self-culture or mixtures of those are also frequently used in studies (Figure 2). The inoculum of selected *Trichoderma* fungi is prepared in the laboratory in sterile plastic Petri dishes with a diameter of 90 mm. PDA medium (16 mL) is placed in each dish (Figure 3). Once solidified, a 5 mm disc of medium that contains mycelium of the relevant isolate is placed in the central part of the dish. The disk is cut out from the 10-day culture. Then, the culture is incubated at 20 °C for three weeks, 20 mL of distilled water is poured onto the sporulating cultures, and the obtained suspension is poured into a flask. A spore suspension of *Trichoderma* isolates is prepared using a three-week-old culture. *Trichoderma* isolates are soaked in 20 mL of sterile distilled water and scraped off with a sterile copper rod. The suspension is filtered and the concentration of *Trichoderma* spores in the mixture is adjusted to a concentration of 10^6^ per ml using a haemocytometer and a light microscope [56,57,58].

## 5. Root Colonization by Fungi of the *Trichoderma* Genus

*Trichoderma* spp. are fungi that are commonly found in soil and root ecosystems. Some strains colonize roots intensively and persistently by penetrating the top layers of the epidermis [59]. Research shows that the intensity of root colonization by fungi of the *Trichoderma* genus varies between species. Andrzejak and Janowska [56] report that in both years of research, among treatments of *Gladiolus hybridus* ‘Advances Red’, in which *Trichoderma* spp. were used, 46.6% and 48.2% of plant roots were colonized by the fungi. A lower percentage of root colonization by *Trichoderma* spp. was obtained by Janowska et al. [57] in *Freesia reflacta* ‘Argentea’ (32.0% and 33.0% in non-illuminated and illuminated plants) and by Andrzejak et al. [58] in *Begonia* × *tuberhybrida* ‘Picotee Sunburst’ (30.5%, 29.5%, and 30.0%, respectively, in plants subjected to late top dressing with Peters Professional Allrounder multi-component fertilizer). Prisa et al. [59] pointed out that the colonization of plant roots with fungi of the *Trichoderma* genus can be very high, as they proved in *Limonium sinuatum* (100.0%). According to Błaszczyk et al. [60], in the rhizosphere, *Trichoderma* spp. colonize the external layers of the roots of herbaceous plants and trees. They also have the ability to penetrate and colonize within roots, or occur as endophytes. These authors used *Triticum aestivum* as an example to demonstrate that a preliminary analysis of morphological, physiological, and metabolic changes indicates that there is no clear-cut plant response to fungi of the *Trichoderma* genus. This may mean that changes taking place in plants depend both on the genus/strain of *Trichoderma* spp. and on the cultivar of the species studied. According to Souza et al. [61], interactions between the plant and the microbiota in the rhizosphere are key factors determining plant health, productivity, and soil fertility. Plant roots synthesize metabolites that are recognized by microorganisms which respond by producing signals that initiate microbial colonization [62]. Plant roots also release sucrose, which is a source of energy to support colonization by microorganisms [63,64]. As mentioned earlier, *Trichoderma* stimulates root growth by producing auxins [13]. During root colonization by *Trichoderma* spp., genes such as ASA1 and MYB77 are induced. In the root, ethylene and auxin can regulate their biosynthetic pathways [65]. According to Stepanova et al. [66], IAA of *Trichoderma* contributes to exogenous auxin-stimulated ethylene biosynthesis through 1-aminocyclopropane-1-carboxylicacid synthase (ACC). In this model, the activity of *Trichoderma* ACC desaturase (ACCD) reduces the availability of ACC necessary for ethylene biosynthesis, and the reduction of ethylene stimulates plant growth through gibberellin (GA) signaling, increasing the degradation of DELLA proteins, which are repressors of GA signaling. Moreover, GAs can control the onset of jasmonic acid (JA) and salicylic acid (SA)-dependent plant defense responses by regulating the degradation of DELLA proteins [65]. Therefore, it seems that defense comes at the expense of growth. To confirm the above, recent studies have indicated new roles of JAZ and DELLA proteins in the regulation of JA-GA coupling, as well as the contradictory relationship between defense and growth. The positive effect of DELLA on JA signaling seems to take place at the level of JAZ repressors, as DELLA proteins interact with JAZ proteins and reduce their ability to repress MYC2 [67,68]. According to Brotman et al. [69], when MYC2 undergoes significant changes, as demonstrated in their studies, during root colonization, growth is promoted through the degradation of DELLAs by GAs, whereas defense is repressed by JAZs repressing MYCs. This shifts the balance towards growth while allowing root colonization by *Trichoderma*.

## 6. The Impact of *Trichoderma* spp. on the Quality of Ornamental Plants

### Plant Height, Number of Shoots, and Leaves

**Figure 4:**
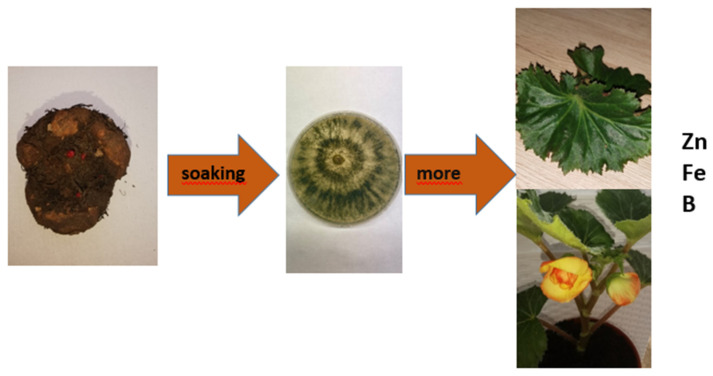
Effect of *Trichoderma* spp. on quality of *Begonia* × *tuberhybrida* ‘Picotee Sunburst’.

Harman et al. [15] claim that *Trichoderma* fungi stimulate the growth of roots, as well as growth in the length and thickness of shoots and leaf surface. However, Lorito et al. [16] indicate that the mechanisms supporting the beneficial effects of plant growth stimulation have not been fully explained and have been based on the suggestion that this stimulation is linked to increased nutrient availability. Andrzejak et al. [58] have demonstrated that fungi of the *Trichoderma* genus do not affect the height and number of shoots in *Begonia* × *tuberhybrida* ‘Picotee Sunburst’, but they do stimulate leaf development in it (Figure 4). In the *Tulipa gesneriana* ‘Golden Parade’, *Trichoderma* spp. Have no impact on the number of leaves, but, depending on the fungus species used, they either stimulate or inhibit leaf blade elongation and influence its width [70]. Using *T. harzianum* T-22 in *Lantana camara* stimulates the elongation and thickening of shoots and the development of leaves [71]. Prisa [72], on the other hand, states that *T. viride* stimulates the elongation and formation of shoots and leaves, as well as the growth of the vegetative mass in plants of three species of the genus *Kalanchoe* (*K*. *pinnata*, *K. tubiflora*, and *K. gastonis-bonnieri*). Moreover, plants treated with *T. viride* show an increased vitamin C content in leaves with greater dry mass (Table 2).

## 7. Flowering of Plants Following the Application of *Trichoderma* spp.

### 7.1. Earliness of Flowering

The earliness of flowering of ornamental plants is a very important parameter that makes it possible to plan harvest for a specific date. Therefore, it is necessary to know how individual species and cultivars respond to the treatments applied to them (Table 2). Research shows that many species of ornamental plants that have *Trichoderma* spp. applied to them tend to flower early. *Trichoderma* spp. make *Freesia reflacta* ‘Argentea’ flower about a week earlier in the winter period without assimilation lighting [57]. This effect is most likely the result of a correctly conducted inoculation of fungi, which involves using a suspension with these fungi to water the substrate placed directly above the tubers. *Trichoderma* are aerobic organisms and thrive best in the surface layers of the substrate [7]. Moreover, Benitez et al. [4] point out that spore formation in fungi of the *Trichoderma* genus occurs faster with increased access to visible light. Moreover, Andrzejak et al. [58] report that using *Trichoderma* spp. can slightly accelerate the flowering of *Begonia* × *tuberhybrida* ‘Picotee Sunburst’ (Figure 4) when the plants are fed with Peters Professional Allrounder multi-component fertilizer at a concentration of 0.2%. However, using a higher concentration of the fertilizer causes the plants to flower 7–8 days earlier. Andrzejak and Janowska [56] write that the earliness of flowering in *Gladiolus hybridus* ‘Advances Red’ depends solely on whether *Trichoderma* spp. are used in their cultivation or not. The authors have demonstrated that, regardless of the year of the research, the flowering of plants grown with fungi of the *Trichoderma* genus starts ten days earlier on average. Cig and Aydion [70] report on the early flowering of *Tulipa* ‘Golden Parade’ following the application of fungi of the *Trichoderma* genus. The authors demonstrated that *T. gamsii* VG47 and *T. harzianum* LO52 accelerated flowering by 2–4 days.

### 7.2. The Quality of Flowers and Inflorescences

In some ornamental plant species, *Trichoderma* fungi impact flower quality traits, such as the length of the peduncle/shoot and the size of flowers (Table 2). Andrzejak and Janowska [56] report that, following the application of *Trichoderma* spp., in *Gladiolus hybridus* ‘Advances Red’, the inflorescence stems growing out of the tubers were longer (by 9.8%), and they ended in longer inflorescences (by 10.0%) with more flowers developing in them (by 12.6%). However, the diameter of flowers in both years of research was not affected by *Trichoderma* spp. treatment. Partially similar results were obtained earlier by Sisodia et al. [74]. The authors demonstrated that applying *Trichoderma* spp. in eight cultivars of *Gladiolus* sp. had a positive impact on the length of the inflorescence and the duration of flowering, but had no influence on the number of flowers. Furthermore, da Cruz et al. [75] report that *Trichoderma* spp. applied in the cultivation of *Gladiolus* ‘Peter’s Pear’ had no influence on the quality of inflorescences, expressed in terms of the length of inflorescence shoot, length of the inflorescence, and the number of flowers. According to the research conducted by Andrzejak et al. [58], *Trichoderma* spp. stimulate the development of buds and flowers, and influence their size in *Begonia* × *tuberhybrida* ‘Picotee Sunburst’. The results they obtained have been confirmed by research conducted by Janowska et al. [57]. The authors report that *Trichoderma* spp. stimulate the development of lateral inflorescence shoots and the development of flowers in *Freesia reflacta* ‘Argentea’, especially in the plants provided with assimilation lighting during cultivation. According to Prisa [73], fungi of the *Trichoderma* genus stimulate flowering in *Pachyphytum oviferum* and *Crassula falcata*.

## 8. Chloroplast Pigment Content in Leaves Following the Application of *Trichoderma* spp.

Chlorophylls are a widespread group of photosynthetic pigments found in higher plants, algae, and cyanobacteria. Chlorophyll is a pigment that plays a key role in the normal course of photosynthesis, in which energy from light is converted into chemical bond energy as a result of the absorption of quanta of light in redox reactions [76]. Therefore, the concentration of chlorophyll in leaves can have a direct influence on the photosynthetic process in a plant [77]. Harman et al. [78] suggested that the improvement of photosynthetic capability in plants, induced by various endophytic *Trichoderma* spp., occurs as a result of an increase in the number of photosynthetic pigments or the expression of genes regulating the biosynthesis of chlorophyll, proteins in the light-harvesting complex, or components of the Calvin cycle. The colonization of crop roots by *Trichoderma* spp. fungi causes greater regulation in genes and pigments that improve photosynthesis in plants. Plants under physiological or environmental stress lose the ability to photosynthesize, as photosystems get damaged, and many cellular processes get disrupted by Reactive Oxygen Species (ROS). Yet, some strains of *Trichoderma* spp. activate biochemical pathways that reduce ROS to less harmful molecules. This and other mechanisms make plants more resistant to biotic and abiotic stresses. Moreover, when the indicators of photosynthesis are increased, more carbon dioxide (CO_2_) gets absorbed from the atmosphere. Carotenoids, on the other hand, are responsible for the stability of lipid membranes, are involved in the accumulation of light during photosynthesis, and in the protection against photooxidation caused by the ROS formed during chlorophyll excitation during photosynthesis [79,80]. The antioxidant effect of carotenoids on lipid membranes depends on their orientation, location, and organization in membranes. Polar and non-polar carotenoids impact on the structure and physiology of tissues in different ways. For example, astaxanthin, which is a polar substance, reduces lipid peroxidation by maintaining a rigid membrane structure [81]. Carotenoids are distinguished by high activity against ROS and free radicals [79].

Most research papers addressing the impact of *Trichoderma* spp. on the content of chloroplast pigments in leaves refer to commercial, edible species [22,23,24]. Yet, a few studies show that the stimulation of the formation of photosynthetic pigments (chlorophyll, carotenoids) by *Trichoderma* spp. applies to ornamental plants too. Andrzejak and Janowska [56] report that the content of chlorophyll a+b and carotenoids in the leaves of *Gladiolus hybridus* ‘Advances Red’ increased significantly following the application of *Trichoderma* spp. (by 66.7% for chlorophyll a+b and by 33.3% for carotenoids). The results obtained indicate that the photosynthetic capability improved in the ‘Advances Red’ cultivar. Andrzejak et al. [58] report that *Trichoderma* spp. stimulate the production of chlorophyll, whose content is reflected by the greenness index, in *Begonia* × *tuberhybrida* ‘Picotee Sunburst’ (Table 2). 

## 9. *Trichoderma* spp. and Plant Nutrition

### 9.1. Macroelement Content in Leaves

According to Yedidia et al. [14], following the application of *Trichoderma* spp., the root system in plants develops better, which provides the plants with access to a larger volume of substrate and promotes better nutrient uptake. It helps these plants win the competition for nutrients against plants with less developed root systems, or in environments with small quantities of mineral compounds. 

According to Altomare et al. [82], phosphorus (P) compounds can be dissolved and retained in the biomass of the *Trichoderma* fungi and released in an accessible form near the roots following the lysis of the mycelium. Research conducted by Andrzejak and Janowska [56] suggest that in the case of *Gladiolus hybridus* ‘Advances Red’, *Trichoderma* fungi have a significant impact on the uptake of potassium (K) and calcium (Ca) by these plants. In both years of the study, the levels of P, K, and Ca found in the leaves of plants treated with *Trichoderma* spp. were significantly higher than in the leaves of control plants. Similar outcomes were obtained by Janowska et al. [57] in the cultivation of *Freesia reflacta* ‘Argentea’. The authors report that *Trichoderma* spp. stimulate the absorption of P and Ca in non-illuminated and illuminated plants of this species. They also stimulate the absorption of K in illuminated plants (Table 2). Ca is a unique macronutrient with diverse, but fundamental, physiological roles in plant structure and signaling [83]. The majority of plant Ca content can be found in the cell walls and in the in the vacuoles; however, it is also a key component regulating the functions of the plasma membrane [84]. Ca additionally controls the activity of various key metabolic enzymes [85].

The benefits of using microorganisms to improve the uptake of nutrients present an opportunity for the latest horticultural practices, as they allow for reducing fertilizer use. The use of biological fertilizers based on microorganisms is an alternative for maintaining high productivity levels and keeping the environmental impact low [83,86,87]. Biological fertilizers can be used as a supplement or alternative to mineral fertilizers in sustainable crop production [87]. Metwally [86] has demonstrated that both arbuscular mycorrhizal (AM) and *T. viride* fungi are compatible with each other and that their combined use is effective not only in improving the biochemical parameters of plants, such as the content of soluble carbohydrates, protein, free amino acids, and acid and alkaline phosphatases, but also in increasing mineral and nutrient content (N, P, K, Ca, Mg, and Zn). 

### 9.2. Microelement Content in Leaves

Micro-nutrients play a key role in the metabolic and physiological processes in plants. It is worth noting that they influence quality [88] more than yield [89]. Micro-nutrients are components of proteins; for example, iron (Fe) is a component of proteins involved in the transport of electrons (ferredoxin), and performs catalytic functions [89]. *Trichoderma* spp. impact the uptake of zinc (Zn), Fe, and boron (B) by *Gladiolus hybridus* ‘Advances Red’ plants [56]. According to Andrzejak et al. [58], in the case of *Begonia × tuberhybrida* ‘Picotee Sunburst’, *Trichoderma* spp. also stimulated the uptake of Zn, Fe, and B (Figure 4). Moreover, Janowska et al. [57] reported that in *Freesia reflacta* ‘Argentea’, *Trichoderma* spp. stimulated the uptake of Fe, manganese (Mn), and Zn in illuminated and non-illuminated plants, whereas illumination and *Trichoderma* spp. stimulated the uptake of copper (Cu) (Table 2). According to Benitez et al. [4], *Trichoderma* spp. are characterized by an ability to rapidly uptake elements found in the rhizosphere in trace amounts. For example, Fe is chelated by *Trichoderma* spp. due to the production of siderophores. Altmore et al. [82], using *T. harzianum isolate* T-22, showed that this isolate facilitates the assimilation of insoluble or poorly soluble elements, such as Fe, Cu, Zn, and Mn, in in vitro conditions, as it increases the solubility of minerals by acidifying the root microenvironment and reducing oxidized metal ions (Fe^3+^, Cu^2+^).

## 10. Conclusions

*Trichoderma* spp. are widely described as plant growth stimulators. This trait tends to be isolate-specific rather than species-specific, and individual isolates show different degrees of plant specificity. Increased root and/or shoot biomass is the most common manifestation of growth stimulation; however, changes in plant morphology and development have been described too. Scientists all over the world conduct research to determine the influence of *Trichoderma* spp. on various groups of plants, mostly crops. However, there is little information on the influence of these fungi on ornamental plants. *Trichoderma* spp. in this group of plants is also an effective biostimulant. *Trichoderma* spp. are important tools in promoting the growth and flowering of ornamental plants. With them, the use of fertilizers can be reduced, thus protecting the environment. The use of *Trichoderma* spp. should be widespread, not only in the cultivation of edible plants, but also in ornamentals.

## Figures and Tables

**Table 1:** *Trichoderma* spp. officially registered as microbial fungicide, crop protection product, and to improve yield in different countries throughout the world.

Product Name	Species of Fungus of the Trichoderma Genus	References
Asperello T34	*T. asperellum*	[46]
Binab TF WP	*T. harzianum* *T. polysporum*	[47]
Esquive WP	*T. atroviride*	[48]
BINAB T	*T. polysporum* *T. harzianum*	[49]
Remedier WP	*T. asperellum* *T. gamsii*	[50]
RootShield^®^-WP	*T. harzianum*	[51]
T-Gro	*T. asperellum*	[52]
Trianum-P	*T. harzianum*	[53]
Trichopel	*T. harzianum*	[54]
Tusal	*T. harzianum* *T. viride*	[55]

**Table 2:** Effect of *Trichoderma* spp. on ornamental plants.

Species	Modifications	References
*Begonia* × *tuberhybrida* ‘Picotee Sunburst’	Earlier flowering; more leaves; more buds and flowers; larger flowers; more chlorophyll; more Zn, Fe, B.	Andrzejak et al. [58]
*Crassula falcata*	More flowers.	Prisa [73]
*Freesia reflacta* ‘Argentea’	Earlier flowering; more side flowering shoots; more flowers; more P, K, Ca, Fe, Mn, Zn, Cu.	Janowska et al. [57]
*Gladiolus* hybridus ‘Advances Red’	Earlier blooming; longer flowering shoots; longer inflorescences; more flowers; more chlorophyll and carotenoids in leaves; more P, K, Ca, Zn, Fe, B.	Andrzejak and Janowska [56]
*Gladiolus* ‘Yellow Jester’, ‘Tiger Flame’, ‘Punjab Morning’, ‘Punjab Dawn’, ‘Pusa Kiran’, ‘Shubhangini’, ‘IIHR’, and ‘Dhanvantri’	Longer flowering, longer inflorescences.	Sisodia et al. [74]
*Gladiolus* ‘Peter’s Pear’	No effect on the quality of flowers and inflorescences.	da Cruz et al. [75]
*Kalanchoe* sp.	Longer and thicker shoots, more leaves.	Prisa [72]
*Lantana camara*	Longer and thicker shoots, more leaves.	Yahya et al. [71]
*Pachyphodium oviferum*	More flowers.	Prisa [73]
*Tulipa gesneriana* ‘Golden Parade’	Earlier flowering, longer leaf blade.	Cig and Aydin [70]

## Data Availability

Not applicable.
